# Subacute Drug-Eluting Stent Thrombosis Caused by Stent Underexpansion: Evaluation by Optical Coherence Tomography

**DOI:** 10.1155/2011/129341

**Published:** 2011-02-23

**Authors:** Roberto Martín-Reyes, Santiago Jiménez-Valero, Felipe Navarro, Raúl Moreno

**Affiliations:** ^1^Interventional Cardiology Unit, Fundación Jiménez Díaz University Hospital, Avenida Reyes Católicos 2, 28040 Madrid, Spain; ^2^Interventional Cardiology Unit, La Paz University Hospital, Paseo Castellana 261, 28046 Madrid, Spain

## Abstract

We present the case report of a patient presenting with ST segment elevation myocardial infarction due to a subacute drug-eluting stent trombosis within the proximal segment of the left circumflex artery (LCX). Six days before a total chronic occlusion was treated at the mid segment of the LCX by overlapping two drug-eluting stents. Optical coherence tomography (OCT) was helpful to demonstrate stent underexpansion of the overlaping segment as the main mechanism of early stent thrombosis. This case is illustrative about the potential role of OCT to identify the mechanisms of ST and thus guiding the PCI procedure. Moreover, our case shows the capability of the Imagewire to cross a severe stenosis due to stent underexpansion that could not be crossed by the IVUS catheter.

## 1. Case Report

**Figure 1:**
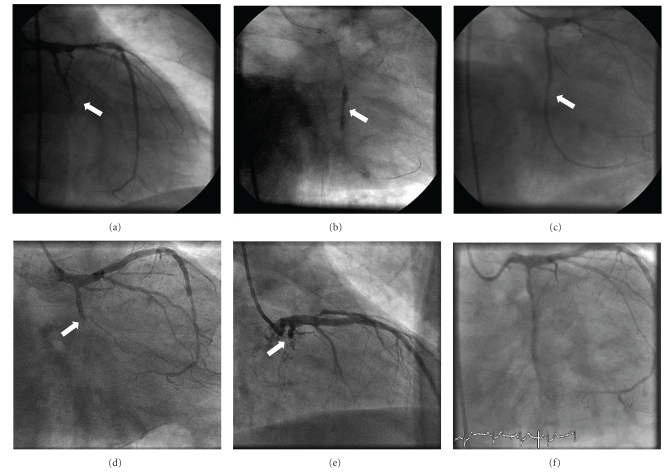
Angiography of the left circumflex artery showing chronic total occlusion in the mid segment (a), postdilatation of the overlap stent segment with a noncompliant balloon (b), and final result showing stent underexpansion in the overlap segment (c). Complete acute occlusion of the left circumflex artery (arrow) due to subacute stent thrombosis ((d) caudal view and (e) cranial view), and the final TIMI-1 flow after failed thrombectomy and progressive dilations of the overlapped segment with oversized noncompliant balloons (f).

A 53-year-old male with a history of arterial hypertension and ex-smoker was admitted into our institution with unstable angina and preserved left ventricular ejection function. Coronary angiography showed multivessel calcified disease with a long calcified lesion in the proximal mid-left descending coronary artery (LAD), chronic total occlusion (CTO) of the mid circumflex artery (LCX) (Figure 1(a)), and a severe lesion in the proximal right coronary artery segment (RCA). The patient decided to be treated by percutaneous revascularization. Initially percutaneous coronary intervention (PCI) of the LCX occlusion was attempted. Using an extra backup (3.5), 6 Fr guide catheter, a PT2 guide wire supported by a 1.5 mm balloon was advanced across the lesion and placed into the distal LCX. The lesion was predilated with 1.5 and 2 × 20 mm Mercury balloons (Boston Scientific Corp., Natick, Massachusetts) and subsequently two overlapping drug-eluting stents (DES) TAXUS Liberte 2.25 × 32 mm and TAXUS Liberte 2.5 × 32 mm (Boston Scientific Corp., Natick, Massachusetts) were deployed in the distal and mid segments, respectively. The angiography showed focal underexpansion in the overlap segment, so, postdilatation using Quantum 2.5 × 12 mm and 2.75 × 12 mm (Figure 1(b), arrow; Boston Scientific Corp, Natick, Massachusetts) noncompliant balloons was performed with poor final angiographic result due to final underexpansion (Figure 1(c), arrow). Two days later, PCI was performed in the LAD and RCA using rotational atherectomy and two DES with good angiographic result. 

**Figure 2:**
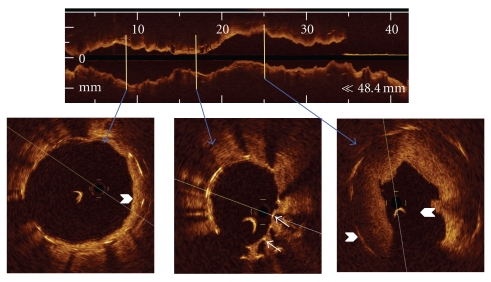
Optical coherence tomographic (OCT) images during pullback from distal (a) to proximal (c) segments. OCT showed severe focal stent underexpansion (b) localized in the overlapping segment, where two struts layers are visible (arrows) and fragments of in-stent remnant thrombus (arrowheads) after balloon dilation.

Six days later, being under dual antiplatelet therapy, the patient presented an acute myocardial infarction with ST segment elevation in posterior leads. Urgent angiography showed in-stent thrombosis of the proximal LCX (Figure 1(d) caudal view and Figure 1(e) cranial view), while both stents in RCA and LAD were patent. Intravenous boluses of aspirin, heparin, and abciximab were administered, and primary PCI was performed. Aspiration thrombectomy was attempted, but the catheter (Export XT, Medtronic, MN, USA) could not be advanced through the overlapping stents segment, even after high-pressure predilation with a 2.5 mm balloon. In order to ascertain the underlying mechanism of stent thrombosis and to guide the intervention, IVUS examination (Atlantis, Boston Scientific Corp., Natick, Massachusetts) was attempted, but, again, the catheter could not be advanced. Then, an optical coherence tomography (OCT) probe (ImageWire, Lightlab Imaging, Westford, Massachusetts) was directly advanced with gentle torque to the distal LCX, and images were obtained using nonocclusive technique, with 2 mm/sec of motorized pullback during automated injection of dye (Iodixanol) through the guiding catheter at 2 mm/sec (Figure 2). OCT revealed severe focal stent underexpansion (ALM 1 mm^2^) localized in the overlap segment with abundant remnant thrombus proximal and distal to the overlap segment. The proximal and distal segments of the stents presented good apposition and expansion. Progressive dilations of the overlapped segment with oversized noncompliant balloons were performed, but were ineffective to improve distal flow (finally, TIMI 1; Figure 1(f)). The patient was discharged 72 hours later without in-hospital complications and preserved LVEF on echocardiogram, with posterolateral hypokinesia.

## 2. Discussion

Stent thrombosis (ST) is a life-threatening complication of PCI, with reported 6-month mortality rates of 29–45% [1]. Drug-eluting stents have reduced the restenosis risk compared with BMS but still remains a 1-2% incidence of stent thrombosis. The aetiology of DES thrombosis is probably multifactorial and has been linked with several factors: withdrawal of antiplatelet drugs, resistance to clopidogrel, clinical features (acute coronary syndromes, renal failure…), and procedural factors, like residual edge dissections, stent underexpansion, or malapposition [2].

OCT is a new imaging technique based on the analysis of backscattered near-infrared light that provides ultra-high image resolution (15 microns) and has demonstrated its utility to identify coronary atherosclerotic plaque composition and guide during PCI procedures [3]. This technique may play an important role to assess the underlying mechanism of ST (stent underexpansion, strut fracture, stent malapposition, edge dissections) [4]. Moreover, OCT is the unique available technique that allows the adequate visualization of the thin neointimal coverage of DES, and may identify incomplete strut coverage in some cases of late ST [5]. Given the ultra-low profile of the image system (ImageWire), similar to a coronary guidewire on its tip, OCT may be useful in cases when IVUS can not cross complex lesions (calcified stenosis, angulated segments, etc.) [6].

Our patient presented an STEMI six days after PCI, and angiography revealed ST of LCX. OCT was helpful to demonstrate stent underexpansion of the overlapping segment as the main mechanism of early ST. This case is illustrative of the potential role of OCT to identify the mechanisms of ST and thus guiding the PCI procedure. Moreover, our case shows the capability of the ImageWire to cross a severe stenosis due to stent underexpansion that could not be crossed by the IVUS catheter.
